# Dr. Edward Clark on Liquid Dovers

**Published:** 1889-03

**Authors:** 


DR. EDWARD CLARK, City Health Physician, writes of McCRAY'S LIQUID DOVERS as follows: "It affords me great pleasure to recommend it (Liquid Dovers) as, without doubt, the most reliable and efficacious form of Dover's Powder yet offered to the medical profession. I have proven, to my own satisfaction, that the results of its judicious administration are something marvelous, . . . and I believe that its trial in all forms of disease where indicated will not result in disappointment."

